# The association between language and recognition of facial emotional expressions in elderly individuals

**DOI:** 10.1590/2317-1782/20212021052en

**Published:** 2022-07-25

**Authors:** Helen Capeleto Francisco, Allan Gustavo Bregola, Ana Carolina Ottaviani, Bruna Moretti Luchesi, Fabiana de Souza Orlandi, Francisco José Fraga, Letícia Pimenta Costa Guarisco, Sofia Cristina Iost Pavarini

**Affiliations:** 1 Programa de Pós-graduação em Enfermagem, Universidade Federal de São Carlos – UFSCar - São Carlos (SP), Brasil.; 2 School of Health Sciences, University of East Anglia – UEA - Norwich, Norfolk, United Kingdom.; 3 Programa de Pós-graduação em Enfermagem. Universidade Federal de Mato Grosso do Sul – UFMS - Campus de Três Lagoas - Três Lagoas (MS), Brasil.; 4 Programa de Pós-graduação em Gerontologia, Universidade Federal de São Carlos – UFSCar - São Carlos (SP), Brasil.; 5 Centro de Engenharia, Modelagem e Ciências Sociais Aplicadas – CECS, Universidade Federal do ABC – UFABC - Santo André (SP), Brasil.

**Keywords:** Elderl, Ageing, Language, Emotions, Facial Expression

## Abstract

**Purpose:**

To check the association between a good performance of language and the recognition of facial emotional expressions in elderly individuals.

**Methods:**

Transversal study performed with 118 elderly individuals from the primary care services of health of a city in the state of São Paulo. Sociodemographic data were collected, regarding the performance of language through the domain of Addenbrooke Cognitive Examination – Revised and Recognition of Facial Emotional Expressions. The sample was divided in thirds according to the performance of language: T1 = the best, T2 = average, and T3 = the worst. The groups T1xT3 were compared regarding the performance of recognition of facial expressions of anger, disgust, fear, happiness, sadness, and surprise, and for the intensities of 40%, 60%, 80%, and 100%. The association of independent variables over the performance of language was analyzed through logistic regression. The multivariate model was built from the results of the univariate analyses and has included the continuous variables by emotion and by intensity. Age and schooling associated to the performance of language in the univariate model were included in the multivariate model in order to adjust association analyses.

**Results:**

The sample was mainly female (84.7%), with an average age of 70.5 years old, and 3.5 schooling years. The variables associated to the best performance of language in comparative analysis of T1 and T3 were: surprise (OR = 1.485, IC 95% 1.194 – 1.846), and disgust (OR = 1.143, IC 95% 1.005 – 1.300).

**Conclusion:**

The recognition of facial emotional expressions of surprise and disgust were shown as important factors associated to the good performance of language.

## INTRODUCTION

The language is an ability that allows the individual to conceptualize, manifest, and interact and communicate, by connecting them to the world. As for the communication, it occurs in several ways, be it through oral or written language, or simply by gestures. So, the communicative efficiency depends not only on the integrity of linguistic skills, but also on the analysis and comprehension of visual components of communication, auxiliary to the pragmatic competence^(1,2)^.

The processing of emotions is an important ability in social interactions and communication, as it allows the individual to identify what the others are feeling, respond properly, avoid conflicts, and regulate their own emotions. On the other hand, deficits in the recognition of emotions may have a negative impact on the social and communicative behavior, promoting difficulties in social interactions^(3,4)^. Thus, the communicative process includes besides the linguistic competences, the processing of emotions, being the facial expression a mediating tool in social interactions, complementary to the analysis and intention of the discourse^(5)^.

The processing of emotions composes an aspect of Social Cognition known by the American Psychiatric Association (APA) and the Diagnostic and Statistical Manual of Mental Disorders (DSM-5) as a criterium for the diagnosis of neurocognitive disorders^(6)^. The social cognition is a dynamic and multifunctional process that requires the simultaneous evaluation of several sources of information, including cognitive (insight, evaluation, self-regulation), internal (autonomous and physiologic responses), social (for instance, type of interaction), and contextual. It aims to understand the capacity of people in noticing the beliefs and intentions of another individual, and also to understand standards, procedures, and social rules, which allow people to live together in society^(4,7,8)^.

The recognition of facial emotional expressions is defined as the capacity of identifying facial emotions in other people, facilitating the inference and interpretation of their actions, sharing feelings, and supporting interpersonal relationships^(9)^. It is related to the behavior, mood, and quality of life of the individual. If some damage occurs during this process, it can favor behavioral alterations and impair the social interactions^(10)^. The processing of emotions can be assessed through the task of Recognition of Facial Emotional Expressions (RFEE), which includes the six basic emotions (happiness, sadness, fear, surprise, anger, and disgust)^(11)^.

One meta-analysis performed in order to investigate the differences of age while identifying facial emotional expressions has shown that the elderly individuals are less precise when asked to identify facial expressions of anger, sadness, fear, surprise, and happiness in comparison with young adults. Nevertheless, the perception of disgust seems to be preserved with ageing, as the performance of elderly individuals was similar to that of younger ones. There was a significant association between the schooling level and the identification of fear and disgust. The authors highlight that the results of this meta-analysis do not support the theory of positivity, which emphasizes that older adults memorize positive emotional expressions better than neutral or negative ones, in relation with younger adults, because the decline in elderly individuals seems to be extended to the positive facial expressions. The authors suggest that brain alterations can explain the pattern observed in elderlies^(12)^.

Contemporaneous Psychological Constructionist Approaches raise the hypothesis that language is an “ingredient” for the generation of perceptions and emotional experiences, thus it is essential for transforming highly vague sensations of pleasure and displeasure into a type of subtle and specific emotion (for instance, while differentiating between anger and fear)^(13)^. It is also supposed that emotional words aid people to store and access the conceptual knowledge about emotions used to give meaning to emotional sensations^(14)^. Besides that, brain regions associated to the processing of language (particularly the semantics processing) are also involved in the processing of emotions, considering that injuries in brain regions related to the language hamper the emotional perception^(13)^.

In ageing, the sustained attention, speed of thinking, and labor memory can be harmed even in the absence of neurodegenerative drugs, being able to interfere in the performance of tasks related to the language, and also to identify emotions^(12)^. Despite the interference of the latest studies in the relationship between emotional processing and language^(15,16)^, we were not able to identify studies in the literature that relate the performance of language with the RFEE task in elderly individuals in the community.

For this study, we started from the hypothesis that there is a relationship between the performance of language and the RFEE performance, since the language allows the conception and identification of emotions through the meaning of words (semantics). Besides that, brain regions associated to the processing of language (particularly the semantics processing) are also involved in the processing of emotions^(13)^. Finally, the objective was to verify the association between the good performance of language and the recognition of facial emotional expressions in elderly individuals.

## METHODS

It is a transversal quantitative study performed with a sample composed by 118 elderly individuals living in the area of the Units of Health of the Family (USF) of a municipality of the state of São Paulo.

All ethical recommendations and care of Resolution 466/2012^(17)^ were met. The research was approved by the Ethical Committee in Research (identification number 1,123,813) of the Federal University of São Carlos (CAAE: 80458017,7,0000,5504). The collect of data started after the reading and signing of the Free and Clarified Term of Consent (TCLE).

The inclusion criteria were used as follows: age ≥ 60 years old and being them registered at one of the USF of the municipality studied. The exclusion criteria were as follows: those who presented difficulty that disabled the performance of the interview, such as severe auditive impairments, history of cerebrovascular accident, alcoholism or use of psychoactive drugs that could jeopardize the comprehension of the instruments of data collection.

The data collect was performed in two stages. In the first one, the interviewers visited the elderlies’ households registered in the USF’s inviting them to join the research. After the verification of the exclusion criteria and signing the Free and Clarified Term of Consent, a sociodemographic characterization questionnaire was applied, having some information collected, such as age (in years), schooling (in years), and sex (male and female). Besides that, the second stage of the interview was scheduled, with a maximum interval of one week between both encounters. In the second stage, cognitive evaluation and recognition of facial expression data were collected. This stage was performed at a predetermined location of their own neighborhood, one of easy access to the participants being ensured a silent, bright, and calm environment. The data was collected between June 2016 and July 2017 by previously trained researchers.

The performance of the language variable was verified from the score of its domain in the Addenbrooke Cognitive Examination – Revised (ACE-R). ACE-R is a protocol of cognitive evaluation that investigates the domains of orientation and attention, memory, oral fluency, language, and visual and spatial skills, scoring from 0 to 100 points. The language is evaluated through tasks of comprehension, reading, writing, repeating, and naming of figures, scoring a total of 26 points^(18,19)^.

RFEE was evaluated through the Emotion Recognition Task (ERT)^(11)^, described by Kessels et al., being it a test presented in the computer with animations of images of facial expressions transformed into short videoclips (dynamic stimuli). The animations transform a neutral face into one with facial expressions at different intensities. The participant observes the facial expression presented on the screen and chooses one among six options of expression (anger, disgust, happiness, surprise, sadness, and fear). Short videos were presented to the individual which include faces of actors of both sexes (two men and two women), changing from neutral to a basic emotion that could have the intensities of 40%, 60%, 80%, or 100%. The framerate of each videoclip varies according to the intensity of the emotion: 0-40% (eight frames), 0-60% (12 frames), 0-80% (16 frames), and 0-100% (20 frames). Similarly, the duration of each video can range from 1 second (0-40%) to 3 seconds (0-100%). After the presentation of the video of 1 to 3 seconds, the image of the face remains on the screen until the responder chooses one answer, with a time limit. In this study, for illiterate participants, the instructions and labels were read for the six emotions and the researcher checked the option chosen by the former, after their confirmation. The presentation started at lower intensities (40%) and followed to the higher ones (100%). 99 videoclips were presented to the participant, being three of them presented to each participant before the start of the test, as a training, having the real test only started after the participant showing their understanding of the task. ERT was exhibited on a computer screen of 14 inches. The total score ranges from 0 to 96 points. For each emotion, the score ranges from 0 to 16 points, and for each intensity 0 to 24 points. The maximum duration of the test is 10 minutes. More information about the faces database, the transformation of facial expressions into animations, and the normative data can be accessed through the original reference^(11)^.

In order to ensure the reliability of the data, depressive symptoms were evaluated according to the Geriatric Depression Scale (GDS-15) with 15 questions and “yes” or “no” answers. The sum of scores obtained was done, being the higher the score, the higher the presence of depressive symptoms. Scores ranging from 0-5 = no depressive symptoms, 6-15 = presence of depressive symptoms^(20)^.

The collected data was inserted and analyzed in the program Statistical Package for Social Science (SPSS), version 21.0. A descriptive statistic was performed by measuring the position and dispersion (average and standard deviation – SD) for the continuous variables and frequency, with percentages (%) for categoric variables in both groups. The normality of variables was checked by Kolmogorov-Smirnov test.

For analyzing the performance of language, the score obtained was organized in decrescent values and the sample divided in thirds, being named T1 the group composed by the third of elderly individuals who presented the best performance (N=49, scores from 22 to 26), T2 the group with average performance (N=27, scores from 17 to 21), and T3, the third with the worst performance (N=42, scores from 0 to 16). For the analyses of this study, groups T1 (best performance) and T3 (worst performance) were used.

After dividing in thirds, the variables of schooling, age, total score at ACE-R, performance of language, disgust, and surprise were maintained as non-parametric, by using the Mann-Whitney test for comparing groups T1 and T3. For the parametric data, test t of Student was used to compare the averages of the other facial emotions, total RFEE between groups T1xT3, and Chi-Square test for categoric variables. The level of significance adopted was 5% (p≤0.05).

In order to verify the association of independent variables over performance of language, univariate and multivariate binary logistic regression analyses were performed. The univariate logistic regression included the independent variables of age, schooling, and GDS (which assess the presence of depressive symptoms) to check the association with the performance of language, being them included in the multivariate model only those with p-value ≤ 0.2. The ACE-R instrument was not included as an independent variable since the classification regarding the performance of language (T1xT3) was also done by using the same instrument, being then colinear variables. The multivariate model was built from the results of univariate analyses, and it included the continuous variables of Total RFEE, by emotion and by intensity, separately. Thus, the variables of age and schooling, which were associated to the performance of language in the univariate model, were considered, so, they have been included in the multivariate model in order to adjust the analyses of association between performance of language and RFEE.

## RESULTS

The total sample was composed by 118 elderly individuals, most of them women (84.7%), ageing from 60 to 91 years old, average of 70.5 (±6.6) years old, and 3.5 (±3.0) schooling years. The demographic characterization of cognitive performance, language, and depressive symptoms in the total sample, as well as the comparative analysis of groups T1 and T3, are presented in Table 1.

**Table 1:** Sociodemographic characterization of performance at ACE-R and the language of the total sample (N=118), and comparison between groups T1 (N=49) and T3 (N=42)

**VARIABLE**	**TOTAL (N=118)**	**T1 (N=49)**	**T3 (N=42)**	**T1 x T3** **p-value**
Schooling, years (average ± SD)	3.54 (± 3.03)	5.31 (± 3.23)	1.71 (± 1.81)	<0.01*
Age, years (average ± SD)	70.47 (± 6,60)	68.67 (± 5.93)	72.62 (± 7.39)	0.01*
Sex, female (%)	84.70	85.70	88.10	0.77**
GDS, points (average ± SD)	3.70 (± 2.72)	3.53 (± 2.65)	3.79 (± 2.93)	<0.01*
Language, points (average ± SD)	1.41 (± 5.19)	23.49 (± 1.78)	12.64 (± 2.70)	<0.01*
ACE-R, points (average ± SD)	62.14 (±16.74)	76.82 (± 11.00)	45.60 (± 8.47)	0.66***

* Mann-Whitney test;

** Chi-square;

*** Test t of Student

**Caption:** SD: standard deviation; T1: group with the best performance of language; T3: group with the worst performance of language; ACE-R: Addenbrooke Cognitive Examination – Revised; GDS: geriatric depression scale

The composition of groups T1 and T3 was similar regarding sex and depressive symptoms, however, group T1 was composed by younger participants, with higher schooling and better cognitive performance (Table 1).

**Figure 1:**
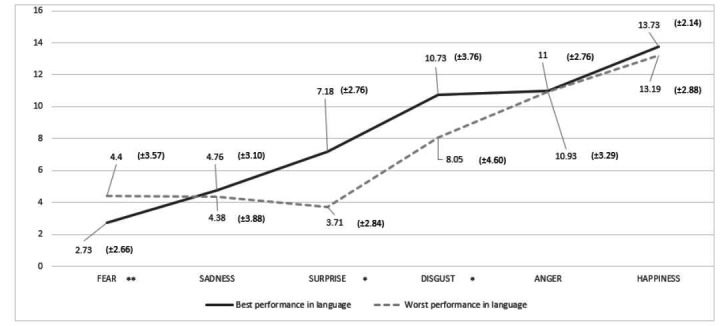
Average of correct answer of groups T1 and T3 at RFEE task by emotion (N=91).

**Figure 2:**
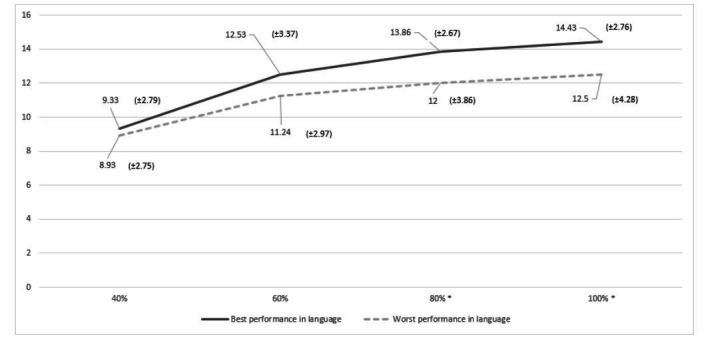
Average of correct answer of groups T1 and T3 at RFEE task by intensity (N=91).

Figure 1 presents a comparative analysis of the average of correct answer of groups T1 and T3 at RFEE task by emotion, while Figure 2 compares the groups at RFEE task by intensity. In both analyses, it is observed a better performance of group T1.

*p=0.02;

**p<0.01

*p<0.05

In the univariate logistic regression analysis, a good performance of language was noted, being it associated to the independent variables of schooling (OR=1.880, p-value<0.000, and IC 1.428 – 2.500), and age (OR=0.967, p-value=0.009, and IC 0.855 – 0.978). GDS, which evaluates the presence of depressive symptoms, was not presented as a predictor of the best performance of language (OR=0.967, p-value=0.661, and IC 0.833 – 1.123).

In conclusion, in order to verify the association between a good performance of language and RFEE, for each emotion and intensity, a multivariate logistic regression was performed including the schooling and age variables in the model to adjust it (Table 2).

**Table 2:** Variables associated to the good performance of language

	**Variable**	**p-value**	**OR** *	**CI95%** *
	Total RFEE	0.128	1.042	0.988-1.099
	Fear	0.078	0.842	0.695 – 1.020
Emotions	**Surprise**	**0.002**	**1.422**	**1.137 -1.779**
	**Disgust**	**0.027**	**1.181**	**1.019-1.369**
	Anger	0.686	1.038	0.867 – 1.171
	Sadness	0.942	1.006	0.862-1.171
	Happiness	0.459	1.090	0.863-1.017
	40%	0.593	1.060	0.855 – 1.315
Intensities	60%	0.352	1.082	0.917-1.277
	80%	0.062	1.176	0.992 – 1.394
	100%	0.128	1.139	0.963-1.348

All emotions and intensities were adjusted by age and schooling

* OR=*Odds Ratio*; CI95% OR = 95% confidence interval for happening by chance

## DISCUSSION

In the present study T1 (best performance of language) is composed by younger individuals, with higher schooling and better cognitive performance without depressive symptoms. As it was consistently noted in the literature, the language suffers influence from ageing, mainly according to the schooling level^(21,22)^.

One study has analyzed the influence of sociodemographic variables such as age, education, gender, and the cultural background over the performance of RFEE. Elderly participants (N = 203; 109 women and 94 men) were submitted to the test and have presented significative effect of ageing and schooling during the performance of RFEE, in such way in which younger individuals with higher schooling level have presented higher scores. There was no difference between genders while performing the test^(23)^. The same fact was observed by researchers^(11)^ who have studied 373 healthy children and adults, between 8 and 75 years old, examining the effects of age, sex, and intellectual power in the perception of emotions. In a metanalysis run in 2018^(12)^ the authors presented a significant association between the schooling level and the identification of fear and disgust. It is interesting to note that in a previous study^(3)^, the emotions of disgust and surprise were the only ones which obtained a similar performance among groups of young and older healthy individuals, what suggests that the emotions of disgust and surprise do not seem to suffer influence of the ageing factor.

Naming the emotions identified in facial expressions is one function of the language influenced by schooling. Researchers have found that participants who concluded a former major education have obtained a higher probability of selecting the correct “name - label” for disgust when compared to those without this university level achieved. According to the authors, the number of correct and incorrect answers is partially influenced by the trend of using certain labels. For example, sadness has a broader meaning for children at kindergarten age than that of university students, which corresponds to the more frequent use of these words by participants without university studies, compared with those from the university^(24)^.

By analyzing the average of correct answers of groups T1 and T3 at RFEE task by emotion, happiness was the one more easily identified in general (13.73 and 13.19 for T1 and T3, respectively), followed by anger (11 and 10.93) and disgust (10.73 and 8.05), making up the highest correct answers rate, superior to 10 points. As for fear, it was the least recognized emotion (4.4 and 2.73 for T1 and T3, respectively), followed by sadness (4.76 and 4.38) with an average performance inferior to five points. The performance for surprise was low for group T3 (score = 3.71), while T1 obtained a superior average (score 7.18), showing a greater facility in noting this emotion in the group with the best performance of language. The literature points out that expressions of fear and surprise can be confused at a fast display^(25,26)^. The functional organization of the system of recognition of facial expression incorporates the distinction between these two emotions, which has been investigated by using functional magnetic resonance in order to explore the activation of different brain regions in response to the expressions of fear and surprise. The researchers have found common mechanisms for both emotions (limbic system, including the amygdala and the parahippocampal gyrus), and that faces of fear promote a higher activation of the systems of attention and memory, whilst surprise results in a higher activation of the system of emotional experience^(25)^. In another study, the facilitator effect of the expression of surprise while differentiating from fear was identified, and this makes the relationship between these two facial expressions closer, having an important adaptative value in which the differentiation between the expression and the sensation of surprise allows the attentional and physiologic preactivation, with a negative bias that would favor a fast defensive response starting even before the stimulus of danger being confirmed, increasing the probabilities of survival of the individual^(26)^. 

The intensity of the emotion is also an important factor for the RFEE task. The best performance was observed at high intensities (80% and 100%) in both groups, being T1 the one with the best performance as the intensity was increased. Regarding the recognition of facial emotions with varying intensities of expressions, studies with elderly individuals have shown that happiness was the easiest emotion to be recognized, and reductions related to the age were identified according to the intensity of sadness, anger, and fear^(3,27)^. Furthermore, by investigating the influence of intensity of the expression in recognizing facial emotions, one study showed that elderlies had a good performance towards happiness, surprise, and disgust, even at a low intensity. Nevertheless, they performed worse than adults in recognizing sadness, anger, and fear, at all intensities^(28)^.

As for the relationship between RFEE and language, the constructionist psychologic method of emotion suggests that words that name concepts of emotion (‘fear’, ‘disgust’, ‘anger’) are in fact constitutive for emotions. In these models, the emotional words support the concept knowledge that helps the brain to provide meaning to the affective sensations in a certain context. By doing that, the concept knowledge helps to ‘build’ emotions, because it transforms ambiguous affective sensations into experiences and perceptions of certain distinct emotions. In the presence of emotional words, some brain regions responsible for semantics processing are activated, such as the prefrontal cortex, hippocampus, and temporal lobe^(13)^. Brain image studies have investigated the emotional effects at lexical, semantic, and morphosyntactic aspects of the language during the comprehension of words and phrases. The evidences analyzed suggest that the emotion is presented in the brain as a set of semantic characteristics in a sensorial, motor, affective, and language network. Besides that, the emotion interacts with several lexical, semantic, and syntactic characteristics in different regions of the brain^(29)^.

Besides the language, the regulation of emotions also represents a fundamental ability for the social interaction^(30)^. So, considering that a good processing of language and the precise recognition of emotions of the interlocutor are fundamental in social interactions, the present study brings contributions by evidencing distinct areas of investigation, such as social and neuropsychologic emotional cognition in the preservation of the language and communication in elderly individuals.

We highlight the transversal delineation as a limitation to the study which disables the analysis of relation of cause and effect, beyond the use of an intentional sample.

As a suggestion for further studies, we highlight the importance of investigating the relation of different aspects of language, such as the pragmatic ones for example with RFEE, besides the influence of contextual hints like the body language and vocal prosody in the recognition of emotions by elderly individuals.

## CONCLUSION

The recognition of facial emotional expressions of surprise and disgust were shown as important factors associated to the good performance of language.
