# Supplementary material for: The association between language and recognition of facial emotional expressions in elderly individuals
Source: Codas. 2022 Jul 25;34(6):e20210052. doi: 10.1590/2317-1782/20212021052en (PMC9886300; doi:10.1590/2317-1782/20212021052en)
Supplement: Translation

# Translation: Associação entre linguagem e o reconhecimento de expressões faciais de emoções em idosos

**DOI:** 10.1590/2317-1782/20212021052pt

**Authors:** Helen Capeleto Francisco, Allan Gustavo Bregola, Ana Carolina Ottaviani, Bruna Moretti Luchesi, Fabiana de Souza Orlandi, Francisco José Fraga, Letícia Pimenta Costa Guarisco, Sofia Cristina Iost Pavarini

**Affiliations:** 1 Programa de Pós-graduação em Enfermagem, Universidade Federal de São Carlos – UFSCar - São Carlos (SP), Brasil.; 2 School of Health Sciences, University of East Anglia – UEA - Norwich, Norfolk, United Kingdom.; 3 Programa de Pós-graduação em Enfermagem. Universidade Federal de Mato Grosso do Sul – UFMS - Campus de Três Lagoas - Três Lagoas (MS), Brasil.; 4 Programa de Pós-graduação em Gerontologia, Universidade Federal de São Carlos – UFSCar - São Carlos (SP), Brasil.; 5 Centro de Engenharia, Modelagem e Ciências Sociais Aplicadas – CECS, Universidade Federal do ABC – UFABC - Santo André (SP), Brasil.

**Keywords:** Idoso, Envelhecimento, Linguagem, Emoções, Expressão Facial

## Abstract

**Objetivo:**

Verificar a associação entre o bom desempenho de linguagem e o reconhecimento de expressões faciais de emoções em idosos.

**Método:**

Estudo transversal realizado com 118 idosos dos serviços de atenção primária à saúde de um município paulista. Foram coletados dados sociodemográficos, de desempenho da linguagem pelo domínio do Exame Cognitivo de Addenbrooke - Revisado e de Reconhecimento de Expressões Faciais de Emoções. A amostra foi dividida em tercis de acordo com o desempenho na linguagem: T1 = melhor, T2 = mediano e T3 = pior. Os grupos T1xT3 foram comparados em relação ao desempenho no reconhecimento de expressões faciais de raiva, nojo, medo, alegria, tristeza e surpresa e para as intensidades 40%, 60%, 80% e 100%. A associação das variáveis independentes sobre o desempenho de linguagem foi analisada por meio de regressão logística. O modelo multivariado foi construído a partir dos resultados das análises univariadas e incluiu as variáveis contínuas por emoção e por intensidade. Idade e escolaridade, associadas ao desempenho de linguagem no modelo univariado, foram incluídas no modelo multivariado para ajustar as análises de associação.

**Resultados:**

A amostra era predominantemente feminina (84,7%), com idade média de 70,5 anos e 3,5 anos de escolaridade. As variáveis ​​associadas ao melhor desempenho de linguagem na análise comparativa de T1 e T3 foram: surpresa (OR= 1,485, IC 95% 1,194 – 1,846) e nojo (OR= 1,143, IC 95% 1,005 – 1,300).

**Conclusão:**

O reconhecimento de expressões faciais das emoções surpresa e nojo mostraram-se importantes fatores associados ao bom desempenho da linguagem.

## INTRODUÇÃO

A linguagem é uma habilidade que permite conceituar, manifestar e interagir e comunicar, conectando o indivíduo com o mundo. Já a comunicação ocorre de múltiplas formas, seja por meio da linguagem oral, escrita ou simplesmente gestual e, por isso, a eficiência comunicativa depende não somente da integridade de habilidades linguísticas, mas também da análise e compreensão dos componentes visuais da comunicação, auxiliares à competência pragmática^(1,2)^.

O processamento de emoções é uma habilidade importante nas interações sociais e na comunicação, pois permite identificar o que os outros estão sentindo, responder adequadamente, evitar conflitos e regular as próprias emoções. Por outro lado, déficits no reconhecimento de emoções podem ter um impacto negativo no comportamento social e comunicativo, gerando dificuldades nas interações sociais^(3,4)^. Assim, o processo comunicativo inclui, além das competências linguísticas, o processamento de emoções sendo a expressão facial uma ferramenta mediadora nas interações sociais e complementar a análise e intenção do discurso^(5)^.

O processamento de emoções compõe um aspecto da Cognição Social, reconhecida pela Associação de Psiquiatria Americana (APA) no Manual de Diagnóstico e Classificação dos Transtornos Mentais (DSM-5) como critério para o diagnóstico de transtornos neurocognitivos^(6)^. A cognição social é um processo dinâmico e multifuncional que requer a avaliação simultânea de várias fontes de informação, incluindo cognitiva (insight, avaliação, autorregulação), interna (respostas autonômicas e fisiológicas), social (por exemplo, tipo de interação) e contextual. Visa entender a capacidade das pessoas de perceber as crenças e intenções de outro indivíduo e de compreender normas, procedimentos e regras sociais, as quais permitem as pessoas conviverem em sociedade^(4,7,8)^.

O reconhecimento de expressão facial das emoções é definido como a capacidade de identificar emoções faciais de outras pessoas, facilitando a inferência e a interpretação de suas ações, compartilhando sentimentos e amparando os relacionamentos interpessoais^(9)^. Está associado ao comportamento, humor e qualidade de vida do indivíduo. Caso ocorra algum dano neste processo, isto pode favorecer alterações comportamentais e comprometer as interações sociais^(10)^. O processamento das emoções pode ser avaliado por meio da tarefa de Reconhecimento de Expressões Faciais de Emoções (REFE), que inclui as seis emoções básicas (alegria, tristeza, medo, surpresa, raiva e nojo)^(11)^.

Uma meta-análise realizada com o objetivo de investigar as diferenças de idade no reconhecimento de expressões faciais de emoção observou que os idosos identificam com menos precisão as expressões faciais de raiva, tristeza, medo, surpresa e alegria em comparação aos adultos jovens. Por outro lado, a identificação de nojo parece preservada com a idade, pois o desempenho dos idosos foi semelhante ao dos mais jovens. Houve associação significativa entre o nível de escolaridade e a identificação do medo e nojo. Os autores apontaram que os resultados dessa meta-análise não se sustentam na teoria da positividade, que enfatiza que adultos mais velhos memorizam informações emocionais positivas melhor do que informações neutras ou negativas, em relação aos adultos mais jovens, pois os declínios em idosos parecem se estender às expressões faciais positivas. Os autores sugerem que alterações cerebrais podem explicar o padrão observado em idosos^(12)^.

Abordagens psicológicas construcionistas contemporâneas levantam a hipótese de que a linguagem é um “ingrediente” na criação de percepções e experiências emocionais, pois é essencial para transformar sensações muito vagas de prazer e desprazer em um tipo de emoção sutil e específica (por exemplo, na diferenciação entre a experiência de raiva e medo)^(13)^. Supõe ainda que as palavras emocionais ajudam as pessoas a armazenar e acessar o conhecimento conceitual sobre emoções usadas para dar significado às sensações emocionais^(14)^. Além disso, regiões cerebrais associadas ao funcionamento da linguagem (em particular o processamento semântico) também estão envolvidas no processamento das emoções, sendo que lesões em regiões cerebrais relacionadas à linguagem prejudicam a percepção emocional^(13)^.

No envelhecimento, a atenção sustentada, velocidade de raciocínio e memória de trabalho podem sofrer prejuízos, mesmo na ausência de doenças neurodegenerativas, podendo interferir no desempenho em tarefas de linguagem e também para identificação de emoções^(12)^. Apesar de estudos recentes inferirem a relação entre processamento emocional e linguagem^(15,16)^, não identificamos na literatura estudos que relacionem o desempenho de linguagem com a tarefa de REFE em idosos da comunidade.

Para esse estudo, partiu-se da hipótese de que há relação entre o desempenho de linguagem e o desempenho de REFE, uma vez que a linguagem, por meio da significação das palavras (semântica), permite a conceituação e identificação das emoções. Além disso, regiões cerebrais associadas ao funcionamento da linguagem (em particular o processamento semântico) também estão envolvidas no processamento das emoções^(13)^. O objetivo foi, portanto, verificar a associação entre o bom desempenho de linguagem e o reconhecimento de expressões faciais de emoções em idosos

## MÉTODO

Trata-se de um estudo transversal e quantitativo, realizado com amostra composta por 118 idosos moradores da área de abrangência das Unidades de Saúde da Família (USF) de um município do interior de São Paulo.

Todas as recomendações e cuidados éticos da Resolução 466/2012^(17)^ foram cumpridos. A pesquisa foi aprovada pelo Comitê de Ética em Pesquisa (número do parecer 1.123.813) da Universidade Federal de São Carlos (CAAE: 80458017.7.0000.5504). A coleta de dados teve início após a leitura e assinatura do Termo de Consentimento Livre e Esclarecido (TCLE).

Utilizou-se como critérios de inclusão: idade ≥ 60 anos e ser cadastrado em uma das USF do município estudado. Os critérios de exclusão foram: apresentar dificuldades que impedissem a realização da entrevista, como dificuldades auditivas graves, histórico de Acidente Vascular Encefálico, alcoolismo ou uso de drogas psicoativas que pudessem comprometer a compreensão dos instrumentos de coleta de dados.

A coleta de dados foi realizada em duas etapas. Na primeira etapa, os entrevistadores visitaram o domicílio dos idosos cadastrados nas USFs, convidando-os a participar da pesquisa. Após a verificação dos critérios de exclusão e assinatura do Termo de Consentimento Livre e Esclarecido, foi aplicado o questionário de caracterização sociodemográfica, sendo coletadas informações como idade (em anos), escolaridade (em anos) e sexo (homem e mulher), além disso, foi agendado um horário para a segunda parte da entrevista, com intervalo máximo de uma semana entre as duas coletas. Na segunda etapa, coletou-se dados de avaliação cognitiva e de reconhecimento de expressões faciais. Esta etapa foi realizada em um local previamente definido no próprio bairro, de fácil acesso para os participantes, sendo assegurado um ambiente silencioso, iluminado e tranquilo. Os dados foram coletados no período de junho de 2016 a julho de 2017 por pesquisadores previamente treinados.

O desempenho na variável linguagem foi verificado a partir da pontuação deste domínio no Exame Cognitivo de Addenbrooke - Revisado (ACE-R). O ACE-R é um protocolo de avaliação cognitiva que investiga os domínios de orientação e atenção, memória, fluência verbal, linguagem, habilidades viso espaciais, com escore geral de 0 a 100 pontos. A linguagem é avaliada por tarefas de compreensão, leitura, escrita, repetição e nomeação de figuras, com pontuação total de 26 pontos^(18,19)^.

O REFE foi avaliado por meio da Tarefa de Reconhecimento de Emoção (ERT)^(11)^, descrito por Kessels et al., que é um teste apresentado no computador com animações de imagens de expressões faciais, transformadas em pequenos videoclipes (estímulos dinâmicos). As animações se transformam de um rosto neutro em um rosto com expressões faciais, em diferentes intensidades. O participante observa a expressão facial apresentada na tela e escolhe uma entre as seis opções de expressão (raiva, nojo, alegria, surpresa, tristeza e medo). Foram apresentados ao indivíduo vídeos de curta duração que incluem uma face de atores de ambos os sexos (dois homens e duas mulheres) que vai do neutro a uma emoção básica que pode ser de 40%, 60%, 80% ou 100% de intensidade. O número de quadros (frames) de cada vídeo clipe varia de acordo com a intensidade da emoção: 0-40% (oito quadros), 0-60% (12 quadros), 0-80% (16 quadros) e 0-100% (20 quadros). Da mesma forma, a duração de cada vídeo pode variar de cerca de 1 segundo (0-40%) a 3 segundos (0-100%). Após a apresentação do vídeo de 1 a 3 segundos, a imagem da face permanece na tela até que o respondente escolha uma resposta, sem limite de tempo. Neste estudo, para os participantes analfabetos, eram lidas as instruções e rótulos verbais das seis emoções e o pesquisador assinalava a opção escolhida pelo participante, após sua confirmação. A apresentação tinha início com intensidades mais baixas (40%) e depois prosseguia para intensidades mais altas (100%). Foram apresentados 99 vídeos clipes ao participante, sendo que três videoclipes eram apresentados a cada participante antes do teste, como forma de treinamento, sendo que o teste só tinha início após o participante demonstrar que havia compreendido a tarefa. O ERT foi exibido em uma tela de computador de 14 polegadas. A pontuação total varia de 0 a 96 pontos. Para cada emoção, a pontuação varia de 0 a 16 pontos e para cada intensidade varia de 0 a 24 pontos. A duração máxima estimada do teste é de 10 minutos. Mais informações sobre o banco de faces, a transformação das expressões faciais em animações e os dados normativos podem ser conferidos na referência original^(11)^.

Para garantir a confiabilidade dos dados, sintomas depressivos foram avaliados por meio da Escala de Depressão Geriátrica (GDS-15), com 15 questões e respostas “sim” ou “não”. Foi realizada a somatória da pontuação obtida, sendo que quanto maior a pontuação, maior presença de sintomas depressivos. Pontuação de 0-5 = sem sintomas depressivos, 6-15 = com sintomas depressivos^(20)^.

Os dados coletados foram inseridos e analisados no programa *Statistical Package for Social Science* (SPSS), versão 21.0. Foi realizada estatística descritiva, com medidas de posição e dispersão (média e desvio-padrão - DP) para as variáveis contínuas e de frequência, com valores percentuais (%) para as variáveis categóricas, em ambos os grupos. A normalidade das variáveis foi verificada por meio do teste Kolmogorov-Smirnov.

Para análise do desempenho de linguagem, a pontuação obtida foi organizada em valores decrescentes e a amostra dividida em tercis, sendo denominado T1 o grupo composto por idosos que compuseram o tercil com melhor desempenho de (N=49, pontuação 22 a 26), T2 o grupo com desempenho mediano (N=27, pontuação 17 a 21) e T3, tercil com pior desempenho (N=42, pontuação 0 a 16). Para as análises deste artigo, foram utilizados os grupos T1 (melhor desempenho) e T3 (pior desempenho).

Após a divisão em tercis as variáveis escolaridade, idade, pontuação total do ACE-R, desempenho de linguagem, nojo e surpresa mantiveram-se não paramétricas, sendo utilizado o teste Mann-Whitney para comparação entre os grupos T1 e T3. Para os dados paramétricos, foi utilizado o teste t de Student para comparação entre as médias das demais emoções faciais, REFE Total entre os grupos T1xT3 e o teste Qui-quadrado para as variáveis categóricas. O nível de significância adotado foi de 5% (p≤0,05).

Para verificar a associação das variáveis independentes sobre o desempenho de linguagem, foram realizadas análises de regressão logística binárias univariadas e multivariadas. A regressão logística univariada incluiu as variáveis independentes idade, escolaridade e GDS (que avalia a presença de sintomas depressivos) para verificar sua associação com o desempenho da linguagem, sendo incluídas no modelo multivariado apenas aquelas com p-valor ≤0,2. O instrumento ACE-R não foi incluído enquanto variável independente uma vez que a classificação quanto ao desempenho de linguagem (T1xT3) também foi feita a partir do mesmo instrumento, sendo, portanto, variáveis colineares. O modelo multivariado foi construído a partir dos resultados das análises univariadas e incluiu as variáveis contínuas REFE total, por emoção e por intensidade separadamente. Assim, considerou-se as variáveis idade e escolaridade, que se mostraram associadas ao desempenho de linguagem no modelo univariado e, portanto, foram incluídas no modelo multivariado para ajustar as análises de associação entre o desempenho de linguagem e o REFE.

## RESULTADOS

A amostra total foi composta por 118 idosos, a maioria mulheres (84,7%), com idades entre 60 e 91 anos e média de 70,5 (±6,6) anos de idade e 3,5 (±3,0) anos de escolaridade. A caracterização demográfica, do desempenho cognitivo, de linguagem e sintomas depressivos na amostra total bem como a análise comparativa nos grupos T1 e T3 são apresentados na Tabela 1.

**Tabela 1:** Caracterização sociodemográfica, de desempenho no ACE-R e na linguagem da amostra total (N=118) e comparação entre os grupos T1 (N=49) e T3 (N=42)

**VARIÁVEL**	**TOTAL (N=118)**	**T1 (N=49)**	**T3 (N=42)**	**T1 x T3** **p-valor**
Escolaridade, anos (média ± DP)	3,54 (± 3,03)	5,31 (± 3,23)	1,71 (± 1,81)	<0,01*
Idade, anos (média ± DP)	70,47 (± 6,60)	68,67 (± 5,93)	72,62 (± 7,39)	0,01*
Sexo, mulher (%)	84,70	85,70	88,10	0,77**
GDS, pontos (média ± DP)	3,70 (± 2,72)	3,53 (± 2,65)	3,79 (± 2,93)	<0,01*
Linguagem, pontos (média ± DP)	18,41 (± 5,19)	23,49 (± 1,78)	12,64 (± 2,70)	<0,01*
ACE-R, pontos (média ± DP)	62,14 (±16,74)	76,82 (± 11,00)	45,60 (± 8,47)	0,66***

* Teste Mann-Whitney;

** Qui-quadrado;

*** Teste t de Student

**Legenda:** DP: desvio padrão; T1: grupos com o melhor desempenho na linguagem; T3: grupo com o pior desempenho na linguagem; ACE-R: Exame Cognitivo de Addenbrooke – Revisado; GDS: sintomas depressivos

A composição dos grupos T1 e T3 foi semelhante no que se refere ao sexo e sintomas depressivos, porém o grupo T1 foi composto por idosos mais jovens, mais escolarizados e com melhor desempenho cognitivo (Tabela 1).

A Figura 1 apresenta uma análise comparativa da média de acertos dos grupos T1 e T3 na tarefa REFE por emoção, enquanto a Figura 2 compara os grupos na tarefa REFE por intensidade. Em ambas as análises, observa-se melhor desempenho do grupo T1.

**Figura 1:** Média de acertos dos grupos T1 e T3 na tarefa REFE por emoção (N=91).

*p=0,02;

**p<0,01

**Figura 2:** Média de acertos dos grupos T1 e T3 na tarefa REFE por intensidade (N=91).

*p<0,05

Na análise de regressão logística univariada verificou-se que o bom desempenho da linguagem está associado às variáveis independentes escolaridade (OR=1,889, p-valor<0,000 e IC 1,428 – 2,500) e idade (OR=0,967, p-valor=0,009 e IC 0,855 – 0,978). O GDS, que avalia presença de sintomas depressivos, não se mostrou um preditor de melhor desempenho da linguagem (OR=0,967, p-valor=0,661 e IC 0,833 – 1,123).

Portanto, para verificar a associação entre o bom desempenho de linguagem e o REFE, para cada emoção e intensidade, realizou-se a regressão logística multivariada incluindo no modelo as variáveis escolaridade e idade para ajustar o modelo (Tabela 2).

**Tabela 2:** Variáveis associadas ao bom desempenho de linguagem

	**Variável**	**p-valor**	OR*	CI95%*
	REFE Total	0,128	1,042	0,988-1,099
	Medo	0,078	0,842	0,695 – 1,020
Emoções	**Surpresa**	**0,002**	**1,422**	**1,137 -1,779**
	**Nojo**	**0,027**	**1,181**	**1,019-1,369**
	Raiva	0,686	1,038	0,867 – 1,171
	Tristeza	0,942	1,006	0.862-1,171
	Alegria	0,459	1,090	0,863-1,017
	40%	0,593	1,060	0,855 – 1,315
Intensidades	60%	0,352	1,082	0,917-1,277
	80%	0,062	1,176	0,992 – 1,394
	100%	0,128	1,139	0,963-1,348

Todas as emoções e intensidades foram ajustadas por idade e escolaridade

* OR = *Odds Ratio*; CI95% OR = 95% intervalo de confiança para a razão de chance

## DISCUSSÃO

No presente estudo, quanto à caracterização dos grupos, o T1 (melhor desempenho de linguagem) foi composto por idosos mais jovens, com maior escolaridade, melhor desempenho cognitivo e sem sintomas depressivos. Assim como apontado consistentemente pela literatura, a linguagem sofre influência do aumento da idade e principalmente do nível de escolaridade^(21,22)^.

Um estudo analisou a influência de variáveis sociodemográficas como idade, educação, gênero e formação cultural no desempenho do REFE. Participantes idosos ​​(N = 203; 109 mulheres e 94 homens) foram submetidos ao teste e apresentaram um efeito significativo do envelhecimento e da escolaridade no desempenho no REFE, de forma que indivíduos mais jovens e com maior escolaridade apresentaram pontuações mais altas. Não houve diferença de gênero no desempenho do teste^(23)^. O mesmo foi constatado por pesquisadores^(11)^ que estudaram 373 crianças e adultos saudáveis, ​​entre 8 e 75 anos, para examinar os efeitos da idade, sexo e capacidade intelectual na percepção emocional. Em uma meta-análise de 2018^(12)^ os autores apontaram uma associação significativa entre o nível de escolaridade e a identificação das emoções de medo e nojo. É interessante notar que em estudo anterior^(3)^, que as emoções nojo e surpresa foram as únicas que tiveram desempenho semelhante entre grupos de jovens e idosos saudáveis, o que nos sugere que as emoções de nojo e surpresa parecem não sofrer influência do fator idade.

Nomear as emoções identificadas nas expressões faciais é uma função da linguagem influenciada pela escolaridade. Pesquisadores identificaram que participantes com ensino superior tiveram maior probabilidade de selecionar o “nome - rótulo” correto para nojo comparados com aqueles sem diploma universitário. De acordo com os autores, o número de respostas corretas e incorretas é parcialmente influenciado pela tendência de usar determinados rótulos. Por exemplo, tristeza tem um significado mais amplo para crianças em idade pré-escolar do que para universitários, o que corresponde ao uso mais frequente dessas palavras por participantes sem educação universitária, em comparação com aqueles com educação universitária^(24)^.

Ao analisar as médias de acertos dos grupos T1 e T3 na tarefa de REFE por emoção, de maneira geral, a alegria foi a emoção mais facilmente reconhecida (13,73 e 13,19 para T1 e T3, respectivamente), seguida de raiva (11 e 10,93) e nojo (10,73 e 8,05) perfazendo as médias de acerto mais altas, superiores a 10 pontos. Já o medo foi a emoção menos reconhecida (4,4 e 2,73 para T1 e T3, respectivamente), seguido da tristeza (4,76 e 4,38) com desempenho médio inferior a cinco pontos. O desempenho na emoção surpresa foi baixo no grupo T3 (pontuação = 3,71), porém no grupo T1 obteve-se uma média superior (pontuação = 7,18), demonstrando maior facilidade na percepção desta emoção no grupo com melhor desempenho em linguagem. A literatura aponta que expressões de medo e expressões de surpresa podem ser confundidas em uma apresentação rápida^(25,26)^. A organização funcional do sistema de reconhecimento de expressão facial incorpora uma distinção entre essas duas emoções, que foi investigada usando ressonância magnética funcional para explorar a ativação de diferentes regiões do cérebro em resposta a expressões faciais medo e surpresa. Os pesquisadores descobriram mecanismos comuns no reconhecimento das duas emoções (sistema límbico, incluindo a amígdala e o giro para-hipocampal) e que rostos com medo provocaram maior ativação em algumas regiões frontais e no giro temporal médio direito, enquanto a ínsula e o córtex pós-central foram amplamente ativados no reconhecimento de rostos surpresos. Esses resultados sugerem que o medo leva a uma maior ativação dos sistemas de atenção e memória, enquanto a surpresa resulta em maior ativação do sistema de experiência emocional^(25)^. Em outro estudo, os autores, identificaram o efeito facilitador da expressão de surpresa na discriminação do medo, e que a estreita relação entre estas duas expressões faciais de emoções tem um importante valor adaptativo, em que a discriminação da expressão e a sensação de surpresa permitiria a pré-ativação atencional e fisiológica, com um viés negativo, que favoreceria uma resposta defensiva rápida, que começaria antes mesmo do perigo do estímulo ser confirmado, aumentando as probabilidades de sobrevivência do sujeito^(26)^.

A intensidade da emoção também é um importante fator para a tarefa de REFE. O melhor desempenho foi observado para as altas intensidades (80% e 100%) em ambos os grupos, sendo que o T1 obteve melhor desempenho que o T3, à medida que aumentava a intensidade das emoções. Com relação ao reconhecimento de emoções faciais com variação nas intensidades das expressões, estudos com idosos mostraram que a alegria foi a emoção mais fácil de reconhecer e verificaram reduções relacionadas à idade na discriminação de intensidade da tristeza, raiva e do medo^(3,27)^. Também investigando a influência da intensidade da expressão no reconhecimento de emoções faciais, um estudo mostrou que idosos tiveram bom desempenho na alegria, surpresa e nojo mesmo em baixa intensidade, no entanto, foram piores que os adultos para reconhecer tristeza, raiva e medo, em todas as intensidades^(28)^.

Sobre a relação entre REFE e a linguagem, o modelo psicológico construcionista de emoção sugere que palavras que nomeiam conceitos de emoção ('medo', 'nojo', 'raiva') são de fato constitutivas das emoções. Nesses modelos, as palavras emocionais apoiam o conhecimento conceitual que ajuda o cérebro a dar sentido às sensações afetivas em um determinado contexto. Ao fazer isso, o conhecimento conceitual ajuda a 'construir' emoções, porque transforma sensações afetivas ambíguas em experiências e percepções de certas emoções distintas. Na presença de palavras emocionais, são ativadas regiões cerebrais responsáveis pelo processamento semântico, como o córtex pré-frontal, hipocampo e córtex temporal^(13)^. Estudos de imagem cerebral investigaram os efeitos emocionais nos aspectos lexicais, semânticos e morfossintáticos da linguagem durante a compreensão de palavras e frases únicas. As evidências analisadas sugerem que a emoção é representada no cérebro como um conjunto de características semânticas em uma rede sensorial, motora, afetiva e de linguagem. Além disso, a emoção interage com várias características lexicais, semânticas e sintáticas em diferentes regiões do cérebro^(29)^.

Além da linguagem, a regulação das emoções também representa uma habilidade fundamental para a interação social^(30)^. Portanto, considerando que um bom funcionamento da linguagem e o reconhecimento preciso das emoções do interlocutor são fundamentais nas interações sociais, o presente estudo traz contribuições evidenciando áreas distintas de investigação, como a cognição social e neuropsicologia emocional na preservação da linguagem e comunicação de idosos.

Apontamos como limitações do estudo, o delineamento transversal, que inviabiliza a análise de relações de causa e efeito, além da utilização de uma amostra intencional.

Como sugestão para novos estudos, destacamos a importância de investigar a relação de diferentes aspectos da linguagem, como os pragmáticos, por exemplo, com o REFE, além da influência de pistas contextuais, como linguagem corporal e prosódia vocal no reconhecimento de emoções pelos idosos.

## CONCLUSÃO

O reconhecimento de expressões faciais das emoções surpresa e nojo mostraram-se importantes fatores associados ao bom desempenho da linguagem.
